# Metabolite profiles evaluated, according to sex, do not predict resting energy expenditure and lean body mass in healthy non-obese subjects

**DOI:** 10.1007/s00394-018-1767-1

**Published:** 2018-07-04

**Authors:** M. Armbruster, M. Rist, S. Seifert, L. Frommherz, C. Weinert, C. Mack, A. Roth, B. Merz, D. Bunzel, R. Krüger, S. Kulling, B. Watzl, A. Bub

**Affiliations:** 1 Department of Physiology and Biochemistry of Nutrition, Max Rubner-Institut, Haid-und-Neu-Str. 9, 76131 Karlsruhe, Germany; 2 Department of Quality and Safety of Fruit and Vegetables, Max Rubner-Institut, Haid-und-Neu-Str. 9, 76131 Karlsruhe, Germany; 3 Institute of Sports and Sports Science, Karlsruhe Institute of Technology, Kaiserstr. 12, 76131 Karlsruhe, Germany

**Keywords:** Resting energy expenditure, Lean body mass, Metabolomics, Human metabolism, Metabolite profiles, Sex differences

## Abstract

**Purpose:**

Differences in resting energy expenditure (REE) between men and women mainly result from sex-related differences in lean body mass (LBM). So far, a little is known about whether REE and LBM are reflected by a distinct human metabolite profile. Therefore, we aimed to identify plasma and urine metabolite patterns that are associated with REE and LBM of healthy subjects.

**Methods:**

We investigated 301 healthy male and female subjects (18–80 years) under standardized conditions in the cross-sectional KarMeN (Karlsruhe Metabolomics and Nutrition) study. REE was determined by indirect calorimetry and LBM by dual X-ray absorptiometry. Fasting blood and 24 h urine samples were analyzed by targeted and non-targeted metabolomics methods using GC × GC–MS, GC–MS, LC–MS, and NMR. Data were evaluated by predictive modeling of combined data using different machine learning algorithms, namely SVM, glmnet, and PLS.

**Results:**

When evaluating data of men and women combined, we were able to predict REE and LBM with high accuracy (> 90%). This, however, was a clear effect of sex, which is supported by the high degree of overlap in identified important metabolites for LBM, REE, and sex, respectively. The applied machine learning algorithms did not reveal a metabolite pattern predictive of REE or LBM, when analyzing data for men and women, separately.

**Conclusions:**

We could not identify a sex independent predictive metabolite pattern for REE or LBM. REE and LBM have no impact on plasma and urine metabolite profiles in the KarMeN Study participants. Studies applying metabolomics in healthy humans need to consider sex specific data evaluation.

**Electronic supplementary material:**

The online version of this article (10.1007/s00394-018-1767-1) contains supplementary material, which is available to authorized users.

## Background

As an outcome of a highly complex biochemical network, resting energy expenditure (REE) is determined by many different endogenous and exogenous factors. The most important exogenous factors are various diseases, climatic conditions, the actual individual stress level, or medication [1, 2]. Amongst endogenous factors, lean body mass (LBM) is the major determinant of REE [3–6]. Furthermore, age, sex, body composition, and biological factors contribute to the variability of REE. Variation in LBM has been shown to explain 65–90% of the between-subject variation in REE [3–6].

LBM is, however, not a homogeneous tissue, but consists of high and low metabolic rate organs, such as visceral organs, brain, and skeletal muscles. Tissue-specific metabolic rates contribute to the observed variation of REE [7]. The body cell mass (BCM) of LBM is the metabolically active mass and is supposed to account for the majority of resting thermogenesis [8]. Therefore, LBM is decisive for human energy expenditure.

The cellular metabolism is reflected in biological matrices such as blood and urine by the sum of its metabolites, the so-called metabolome. Recent progress in high-throughput analytical technologies and bioinformatics now permits simultaneous analysis of hundreds of small metabolites constituting the metabolome. Metabolomics is already widely used in medicine mainly to identify biomarkers for metabolic diseases [9]. Additionally, it is used in nutrition research to identify dietary biomarkers, in studies of diet-related diseases, in dietary intervention studies as a tool to identify molecular mechanisms [10], and furthermore, in exercise science to understand the metabolic responses to physical stress [11].

Metabolomics (blood, urine) can accurately distinguish age and sex differences in humans [12–19], but can only partially explain the components of body composition [20–23] such as LBM, muscle and fat mass. It remains unclear whether the discrimination of LBM is nothing more than the discrimination of sex, because most of the metabolites for sex seem to be identical to the metabolites discriminating LBM [24]. To date, limited data are available from metabolomics studies which determined predictors of REE. Results from a cancer patient study revealed that none of the algorithms applied to 63 urine metabolites could predict variations in REE [24]. Whether the REE of healthy humans is associated with distinct metabolite profiles has not been investigated so far.

Therefore, our aim was to investigate in a cross-sectional study the association of REE and LBM, respectively, with metabolite patterns in plasma and 24-h urine of healthy men and women. More than 1000 analytes from a wide range of chemical classes were obtained through the combination of non-targeted GC × GC–MS, different targeted GC–MS and LC–MS methods, as well as ^1^H-NMR. In this cohort, we recently identified distinct metabolite patterns associated with age and sex [19]. Since sex has an impact on LBM, which is the major determinant of REE, we particularly considered these factors during data evaluation.

## Methods

### Study design and subjects

The Karlsruhe Metabolomics and Nutrition (KarMeN) study is a cross-sectional study that was performed at the Max Rubner-Institut (MRI) in Karlsruhe, Germany, between 2011 and 2013 and was described in detail by Bub et al. [25].

After medical examination, 301 healthy, normal weight and overweight adults (129 females, 172 males) 18–80 years of age were included into the study. All subjects were non-smokers and took no medication known to influence energy metabolism or body composition. Exclusion criteria included the presence of acute or chronic illness and pregnancy or breastfeeding. Additionally, if a participant did not follow the study protocol, their results were excluded. In females, all measurements were performed during the luteal phase of the menstrual cycle to reduce cycle-related impact on metabolites and to avoid blood contaminating urine samples.

Following a 10-h overnight fast, body weight and height were measured following standardized operation procedures: weight was measured without shoes, with light clothing/underwear, and after voiding at an accuracy of 0.1 kg, height to the nearest 0.5 cm with a stadiometer (seca, Hamburg, Germany).

REE was measured by indirect calorimetry (IC) using a ventilated hood system (Vmax Encore 29 n, SensorMedics BV, Bilthoven, The Netherlands). In accordance with the recommended best practice guidelines [26], the subjects lay down in comfortable beds in a quiet special study room. Room temperature was 22–24 °C at a constant humidity. The IC measurement started with an initial 10-min period to accustom the participant to the device and test conditions. Subsequently, a 20-min recording period followed under strict resting conditions. Flow calibration was performed by a 3-L syringe, and gas analyzers were calibrated before. Data were collected every 20 s, and acquired volume of oxygen (*V*O_2_) and carbon dioxide (*V*CO_2_) were converted to REE (kcal/24 h) using the equation of Weir (3.940 × *V*O_2_ + 1.106 × *V*CO_2_) × 1440 − 2.17 × N_2_ (*V*O_2_ and *V*CO_2_ measured in ml/min; N_2_: nitrogen in g/24 h) [27]. Nitrogen excretion was measured from 24-h urine collections.

Body composition was assessed by dual-energy X-ray absorptiometry (DXA) (Lunar iDXA™ GE Healthcare, USA) immediately after the IC and standard anthropometry measurements. For the analysis of fat mass, bone mass, and LBM enCORE Software v16 was used. Appendicular LBM is the sum of Arm and leg LBM. Trunk LBM was calculated by subtracting appendicular LBM from whole body LBM.

### Urine and plasma sampling

The day before examinations the participants collected their urine over a period of 24 h. Collection bottles were kept in cool bags with cooling units throughout. The completeness of the 24-h urine collection was verified using the PABA method (para-aminobenzoic acid) through HPLC-UV by the method of Jakobsen et al. [28]. Upon delivery of the 24-h urine samples in the study center, the volume was recorded. Subsamples were centrifuged at 1850×*g* at 20 °C and aliquots were stored at − 196 °C until analysis.

Blood samples were collected from an antecubital vein into 9-mL EDTA plasma tubes (S-Monovette, Sarstedt, Nümbrecht, Germany). Blood was centrifuged at 1850×*g* at 4 °C and aliquoted into small portions. In addition, serum samples (S-Monovette Z-gel, Sarstedt, Nümbrecht, Germany) were collected for standard clinical biochemistry analyses. All samples were initially frozen at − 20 °C for 1 day and then cryopreserved at − 196 °C until analysis. Quality control (QC) samples were prepared by pooling fasting plasma samples and 24-h urine samples, respectively, from KarMeN participants. These QC samples were used for all analytical methods applied.

### Metabolomic analyses

To obtain a preferably broad coverage of the metabolome of human biofluids, a number of different targeted and non-targeted analytical methods were applied. This section provides a short overview of the different analytical methods used. Details are available in the supplement of Rist et al. [19]

#### Non-targeted GC × GC–MS analysis of plasma and urine samples

All 24-h urine and fasting plasma samples were analyzed by non-targeted GC × GC–MS using a Shimadzu GCMS QP2010 Ultra instrument equipped with a ZOEX ZX2 modulator according to the method established by Weinert et al. [29]. With this method a wide range of metabolites can be detected, such as amines, amino acids, organic acids, sugars, sugar alcohols, other polyols etc.

#### Semi-targeted GC–MS analysis of sugar species in urine samples

As some isomeric sugar species cannot be sufficiently resolved with the non-targeted GC × GC–MS approach [29] but may play an important role in human metabolism, a complementary targeted GC–MS sugar profiling method was developed for urine samples using a Shimadzu GCMS QP2010 Ultra instrument. Overall 66 metabolites, consisting of 40 known sugar species, 15 unknown sugar species, and 11 non-sugar-compounds were detected with this method.

#### Targeted GC–MS analysis of fatty acids in plasma

The chromatographic separation of plasma fatty acids usually requires the application of specialized polar columns and can thus not be done adequately using a standard apolar × medium-polar GC × GC column setup. For this reason, we used the method described by Ecker et al. [30] with minor modifications to determine plasma fatty acids as methyl esters. Using a GC single quadrupole instrument (Shimadzu GCMS QP2010 Ultra) and a BPX90 column (Trajan Scientific), 48 fatty acids could be determined in plasma.

#### LC–MS metabolite profiling using the Absolute IDQ™ p180 kit

Acyl carnitines, amino acids, biogenic amines, phosphatidylcholines, and sphingomyelins were determined by LC–MS in fasting plasma samples using the Absolute IDQ™ kit developed by Biocrates AG (Innsbruck, Austria) [31].

#### Targeted LC–MS analysis of methylated amino compounds

A targeted quantification UPLC–MS/MS method for seven amino compounds in plasma, including L-carnitine, choline, and trimethylamine-N-oxide (TMAO) was established using an Acquity UPLC H-Class system coupled to a Xevo TQD triple quadrupole MS (both from Waters, Eschborn, Germany) [32].

### Targeted LC–MS analysis of bile acids

Analyses of 14 bile acids were done from fasting plasma using a 1200 series HPLC system (Agilent, Waldbronn, Germany) coupled to a Q-Trap 3200 mass spectrometer (AB Sciex, Darmstadt, Germany) as described in Frommherz et al. [33].

#### Non-targeted NMR analysis of plasma and urine samples

All plasma and urine samples were analyzed by 1D-^1^H-NMR spectroscopy as described in Rist et al. [19, 34]. Typically, metabolites that can be detected include organic acids, amino acids, amines, sugars, sugar alcohols, and others.

#### Standard clinical biochemistry

Calcium, chloride, potassium, sodium, and phosphate concentrations were determined in a 24-h urine specimen. Due to potential interferences with metabolomics analyses, urine had not been acidified by hydrochloric acid. Calcium, chloride, potassium, sodium, phosphate, and also iron concentrations, as well as bilirubin, LDL-, HDL-, and total cholesterol, triglycerides, glucose, uric acid, urea, free T3, and free T4 thyroid hormone concentrations were determined in blood serum. Analyses were carried out by the medical laboratory MVZ Labor PD Dr. Volkmann und Kollegen GbR (Karlsruhe, Germany) which is an accredited lab according to DIN EN ISO 15189:2001, using standard analytic procedures. Creatinine was quantified in 24-h urine specimens using a photometric assay based on the Jaffé reaction (DetectX^®^ Urinary Creatinine Detection Kit; Arbor Assays, Ann Arbor, MI, USA). Total urinary nitrogen was quantified by the Kjeldahl method [35, 36]. 25-hydroxyvitamin D and its epimer were quantified by an in-house LC–MS/MS method using serum calibrators and controls from Chromsystems (Gräfelfing, Germany).

### Data processing

#### (GC×)GC–MS

GC × GC–MS raw data files were processed by non-targeted alignment with in-house developed R-modules as described recently [37]. Signal intensity drift, i.e., intra- and inter-batch effects occurring during the 4–5 week measurement period were corrected by means of regularly injected quality control (QC) samples [38–40]. For the data of the semi-targeted GC–MS analysis of sugar species in urine, an automatic method for integration was prepared using the Postrun Analysis feature of GCMSSolution (v 4.1.1.). An excel table with integrated peak areas of the chosen substances was made for further data processing.

#### LC–MS metabolite profiling (Absolute IDQ™ p180 kit)

To analyze the samples of the entire study, five Absolute IDQ well plates were used. To account for possible batch effects between the plates, data normalization as described in the manufacturer’s user manual was applied based on the pooled QC samples, which were extracted and measured ten times on each well plate in between study samples.

#### NMR

All spectra were automatically phased with the Bruker AU program apk0.noe. Using the programme AMIX (v 3.9.14.) (Bruker, Rheinstetten, Germany), plasma spectra were then referenced to the EDTA signal at 2.5809 ppm and bucketed graphically, such that buckets wherever possible contained only one signal or group of signals and no peaks were split between buckets. Urine spectra were resampled to bring them to a uniform frequency axis. Then, spectra were aligned by “correlation optimized warping” [41] and bucketed using an in-house developed software based on Python, again trying to define buckets that contain only one signal or group of signals and not splitting peaks between buckets whenever possible. Identification of metabolites important for prediction of REE, LBM, or sex was achieved with Chenomx NMR Suite 8.1 (Chenomx, Edmonton, Canada).

### Data analysis

Data of the different analytical platforms were integrated into a common data matrix, consisting of 301 samples and > 1000 analytes (including knowns and unknowns). Analytes with a detected frequency lower than 75% in the study samples were eliminated from the data matrix prior to statistical analysis. Non-detected values were replaced by values corresponding to 1/10 × limit of quantitation (LOQ) in targeted methods, where no limit of detection (LOD) was determined; 1/2 × LOD in methods, where LOD was determined/available, 1/2 × minimal intensity for non-targeted MS-based methods.

The columns of this common data matrix were mean centered and scaled by standard deviation prior to analysis. This transformation leads to a uniform scale (mean = 0, SD = 1) for all analytes so that they are comparable between analytical platforms. Using the raw values without scaling would put more weight on the platform that produces the highest absolute values [42]. The resulting matrix was used as input for three different prediction models [support vector machine (SVM) with linear kernel, generalized linear model net (glmnet), and partial least squares (PLS)]. The prediction performance of these models is dependent on model specific hyperparameters which have to be optimized. For example, SVM uses a cost parameter C that controls the trade-off between complexity of the decision function and training error. In glmnet, parameters *α* and *λ* are tuned, and in PLS, the number of components (ncomp) is tuned. To find the optimal value for the hyperparameter, a grid search in conjunction with a nested 5 × 10-fold cross-validation (CV) scheme [43] was applied, and the average of the resulting 50 values was used in the final model.

REE, LBM and sex were treated as dependent variables in the prediction models. Metabolites from targeted and non-targeted metabolomics methods (GC × GC–MS, GC–MS, LC–MS, and NMR) as well as standard clinical biochemistry were used as independent variables. When the model was used for continuous outcomes, the root mean squared error (RMSE) and *R*^2^ were calculated to estimate performance of the predictions. Otherwise, each of these algorithms uses a labeled data set to produce a classifier that can predict the class label of a new person. Here, classes were defined as tertiles (lowest, middle, and highest tertile). Tertiles of REE (and LBM) were treated as dependent variables in the prediction models. Based on the data matrix, the algorithms tried to classify subjects to the highest or lowest tertile of REE (and LBM), respectively. When the model was used to predict sex or categorically considered REE (or LBM), the classification accuracy was assessed.

Finally, we showed a ranking of the top 20 analytes in the metabolite patterns of REE, LBM, and sex. Therefore, analytes were assigned a rank for each algorithm according to their weight, the ranks of the three algorithms were averaged, and analytes sorted according to mean rank. A big advantage of the mean rank is that important metabolites to predict REE, LBM and sex could be identified by each of the different machine learning algorithms. Therefore, metabolites that were important in all three algorithms may be considered to be biologically relevant.

Identification of unknown substances from non-targeted analyses that are important for the prediction of sex or age was performed by comparison with databases, as described in Weinert et al. and Egert et al. [29, 37] for GC × GC–MS or with the Chenomx NMR Suite 8.1 (Chenomx, Edmonton, Canada) for NMR. Statistical analyses were performed with SAS (version 9.4, SAS Institute, Cary, NC, USA) and the statistical software ‘R’ (version 3.2.2) with the R package ‘caret’, version 6.0-71.

## Results

After quality checks and data filtering, 442 plasma analytes were included in the metabolomics data analyses. Of these, 174 were derived from targeted analyses and thus known a priori. Of the detected analytes from non-targeted analyses, approximately 30% could be identified by comparison with databases. For 24-h urine samples, 531 analytes were included in the analyses after data filtering. Targeted analyses contributed 57 a priori known metabolites, whereas from the non-targeted analyses approximately 20% of analytes could be identified. A full descriptive table of the metabolites that were identified and quantified is shown in Rist et al. [19].

### Participant characteristics

Basic characteristics of the KarMeN study participants as well as selected anthropometric, physiological, and functional parameters assessed are listed in Table 1. According to the study criteria, participants were healthy and showed clinical parameters within the reference values. For the participants of the KarMeN study, LBM (*R*^2^ = 0.77; linear regression) as well as trunk (*R*^2^ = 0.76) and appendicular LBM (*R*^2^ = 0.77) are associated with REE, while trunk to appendicular LBM ratio showed minor associations (*R*^2^ = 0.38). REE and LBM were significantly higher in men compared to women (Table 1). Therefore, the calculation of the metabolite patterns of REE and LBM, respectively, was performed separately for men and women. Additionally, we adjusted REE for LBM by univariate linear regression analysis with REE as dependent and LBM as independent variable. The residuals of the model represent the LBM adjusted REE and show no significant differences between men and women.

**Table 1:** Basic anthropometric characteristics of the study participants

	Men (*n* = 172)	Women (*n* = 129)	Total (*n* = 301)
Age (years)	44.4 ± 17.9*	51.7 ± 15.0	47.5 ± 17.1
Height (cm)	180.1 ± 7.2*	166.8 ± 6.5	174.4 ± 9.5
Weight (kg)	79.2 ± 10.2*	64.4 ± 8.3	72.9 ± 11.9
BMI (kg/m^2^)	24.4 ± 2.7*	23.2 ± 2.9	23.9 ± 2.9
LBM (kg)	61.2 ± 6.9*	42.6 ± 3.9	53.2 ± 10.9
Trunk LBM (kg)	32.2 ± 3.4*	23.6 ± 2.1	28.5 ± 5.2
Appendicular LBM (kg)	29.0 ± 3.7*	18.9 ± 2.1	24.7 ± 5.9
Fat body mass (kg)	18.6 ± 6.7*	22.0 ± 6.7	20.1 ± 6.9
Body fat (%)	22.9 ± 6.4*	33.5 ± 6.7	27.4 ± 8.4
REE (kcal/day)	1574 ± 191*	1194 ± 127	1411 ± 251
Waist circumference (cm)	87.8 ± 8.9*	79.1 ± 8.3	84.1 ± 9.7
Blood pressure systolic (mmHg)	128.3 ± 14.1*	120.7 ± 18.1	125.1 ± 16.3
Blood pressure diastolic (mmHg)	84.8 ± 10.6	83.8 ± 12.4	84.4 ± 11.4

Data are given as mean ± SD

*p < 0.001 (*t* test) between men and women

### Metabolite profiles of resting energy expenditure in plasma and urine

The metabolite pattern of REE was calculated separately for women and men, in plasma and urine, respectively. In the first step, REE was used as a continuous variable (Table 2); in the second step REE classified into tertiles was considered (Table 3). No significant associations between urine or plasma metabolites with REE could be identified using any of the three models (SVMlinear, glmnet, and PLS) in healthy subjects. In the tertile classified approach (Table 3), the most accurate predictor in men was 64.4% accuracy (PLS in urine) and 62.4% accuracy (PLS in plasma) for women. Accuracy would be expected to be 50% based on random numbers.

**Table 2:** Prediction of REE of the KarMeN study participants (separately for women and men) based on metabolite profiles in plasma and urine using different algorithms

Matrix	Algorithm	Men (*n* = 172)		Women (*n* = 129)	
		RMSE	*R* ^2^	RMSE	*R* ^2^
Plasma	SVM	184	0.020	125	0.008
	glmnet	172	0.147	131	− 0.085
	PLS	186	− 0.007	140	− 0.263
Urine	SVM	187	− 0.019	127	− 0.044
	glmnet	179	0.072	128	− 0.069
	PLS	186	− 0.025	140	− 0.298

**Table 3:** Prediction of REE tertiles of the KarMeN study participants (separately for women and men) based on metabolite profiles in plasma and urine using different algorithms

Matrix	Algorithm	Prediction accuracy %		
		Total	Low	High
Plasma				
Men (*n* = 113)	SVM	60.5	59.7	61.2
	glmnet	62.8	63.4	62.3
	PLS	62.2	65.2	59.5
Women (*n* = 81)	SVM	60.8	62.3	59.0
	glmnet	61.2	63.5	59.0
	PLS	62.4	62.3	62.5
Urine				
Men (*n* = 115)	SVM	64.2	65.7	63.0
	glmnet	64.2	67.8	61.2
	PLS	64.4	68.4	60.7
Women (*n* = 86)	SVM	59.0	56.8	60.8
	glmnet	57.5	54.6	60.2
	PLS	59.1	59.1	58.8

### Metabolite profiles of lean body mass in plasma and urine

Since LBM is the major determinant of REE, we applied predictive modeling to investigate whether a metabolite pattern exists for LBM. Calculations were performed based on plasma and urine data for women and for men. In healthy subjects, no significant associations between either urine or plasma metabolites and LBM could be identified using the three models (SVMlinear, glmnet, and PLS) (see Supplemental Tables 1 and 2).

The calculation of the metabolite patterns of REE and LBM for men and women separately showed no meaningful results. It is known from other studies that age and sex differences exist in the human plasma and urine metabolome [12–18]. Nevertheless, if and to which extent the discrimination of sex is responsible for the discrimination of body composition, has not been clarified definitely [20–23]. Furthermore, to which extent sex is important in the prediction of REE still remains unknown. Therefore, the following calculations were performed for both sexes together. In addition, the results were compared with the prediction of sex differences.

### The impact of sex on REE- and LBM-associated metabolite profiles

To compare predictions of REE and LBM with the prediction of sex, predictive models were calculated by combining male and female data sets. Prediction accuracy in the study group was excellent for sex (SVM accuracy = 98.1%), for REE (SVM accuracy = 93.4%), and for LBM (SVM accuracy = 98.3%) in plasma (see Supplemental Table 3). When adjusting REE for LBM, these metabolites important for the correct prediction of either sex, REE, or LBM groups showed a high degree of agreement (Table 4). 17 of the top 20 metabolites in urine (12 of the top 20 metabolites in plasma) for REE were identical, with the metabolites discriminating LBM. For REE and sex, 13/20 metabolites in urine and 11/20 in plasma were identical. A similar result was found for the agreement between LBM and sex: 12/20 metabolites in urine and 14/20 in plasma were identical. Here, we present mean ranks of metabolites which were slightly different, but showed an overlap between the methods SVM, glmnet and PLS. As an example individual ranks from SVM, glmnet, and PLS of most important urine metabolites in predicting REE (tertile classified approach) are presented in Supplemental Table 6.

**Table 4:** Top 20 metabolites of the 301 KarMeN study participants for the accurate prediction of REE, LBM, and sex in urine and plasma, respectively (REE and LBM: men and women considered together)

Matrix	Rank	REE	LBM	Sex
Urine	1	U 2.19 (NMR)^1^	Creatinine 2 (NMR)^b^	4-DTA (GC × GC)^a,b^
	2	Citrate 2 (NMR)^a^	4-DTA (GC × GC)^b^	α-KGA (GC × GC)^a,b^
	3	4-DTA (GC × GC)^a^	α-KGA (GC × GC)^b^	Citrate 2 (NMR)^a,b^
	4	U 7.57 (NMR)^a^	Citrate 2 (NMR)^b^	Creatinine 2 (NMR)^a,b^
	5	Citrate 1 (NMR)^a^	3-Indoxyl sulfate (NMR)^b^	*N*-AAA (GC × GC)
	6	Creatinine 2 (NMR)^a^	U 2.19 (NMR)^b^	U 2.19 (NMR)^a,b^
	7	U 05 (GC)	Citrate 1 (NMR)^b^	U 3.93 (NMR)^a,b^
	8	U 3.93 (NMR)^a^	U 3.93 (NMR)^b^	U 0566 (GC × GC)^a,b^
	9	U 0566 (GC × GC)^a^	U 0566 (GC × GC)^b^	U 7.57 (NMR)^a,b^
	10	4-DTA (NMR)^a^	U 7.57 (NMR)^b^	Citrate 1 (NMR)^a,b^
	11	3-Indoxyl sulfate (NMR)^a^	Creatinine 1 (NMR)^b^	4-DTA (NMR)^a^
	12	α-KGA (GC × GC)^a^	U 05 (GC)	3-Methylglutaric acid^c^ (GC × GC)
	13	Citrate (GC × GC)	U 0599 (GC × GC)	l-Cysteine (GC)
	14	PAA (GC × GC)	Leucine (NMR)^b^	3-Indoxyl sulfate (NMR)^a,b^
	15	Hippuric acid (NMR)	Gluconic acid (GC)	Creatinine 1 (NMR)^a,b^
	16	Gluconic acid (GC)	PAA (GC × GC)	4-HPPA (GC × GC)
	17	*cis*-Aconitate (NMR)	Creatinine (CB)	Leucine (NMR)^a,b^
	18	U 4.30 (NMR)	Creatinine (GC)	d-Fructose isomer 2 (GC)
	19	Creatinine 1 (NMR)^a^	*cis*-Aconitate (NMR)	d-Fructose isomer 1 (GC)
	20	Leucine (NMR)^a^	Citrate (GC × GC)	U 0704 (GC × GC)
Plasma	1	Creatinine (CB)^a^	Creatinine (CB)^b^	Creatine (NMR)^a,b^
	2	Uric acid (CB)^a^	Uric acid (CB)^b^	Creatinine (CB)^a,b^
	3	U 2.91 (NMR)^a^	Creatine (NMR)^b^	U 2.91 (NMR)^a,b^
	4	Creatinine 1 (NMR)^a^	SM C16.1 (Biocrates)	Phosphate (CB)^b^
	5	SM C18.1 (Biocrates)^a^	Creatinine 1 (NMR)^b^	Creatinine (Biocrates)^a,b^
	6	4-HPLA (GC × GC)	Creatinine (Biocrates)^b^	Creatinine 1 (NMR)^a,b^
	7	SM C16.1 (Biocrates)	U 2.91 (NMR)^b^	Uric acid (CB)^a,b^
	8	Creatinine (Biocrates)^a^	Leucine 1 (NMR)^b^	U 3.66 (NMR)^b^
	9	Sarcosine (LC)^a^	U 0.81 (NMR)^b^	Glycine (NMR)^b^
	10	Creatine (NMR)^a^	LysoPC.a.C20.4 (Biocrates)	SM C18.1 (Biocrates)^a,b^
	11	U 1.08 (NMR)	Glycine (NMR)^b^	U 0.81 (NMR)^a,b^
	12	U 0.81 (NMR)^a^	U 3.66 (NMR)^b^	Creatinine 2 (NMR)
	13	U 1.09 (NMR)	U 1.26 (NMR)^b^	U 1.26 (NMR)^b^
	14	Isoleucine (Biocrates)	Phosphate (CB)^b^	U 3.37 (NMR)
	15	SM C18.0 (Biocrates)	SM C18.1 (Biocrates)^b^	Proline (NMR)^a^
	16	Valine (NMR)	LysoPC.a.C20.3 (Biocrates)	PC.aa.C32.2 (Biocrates)
	17	Proline (NMR)^a^	Valine (NMR)	Sarcosine (LC)^a^
	18	Leucine (Biocrates)	U 3.33 (NMR)^b^	Leucine 1 (NMR)^a,b^
	19	Leucine 1 (NMR)^a^	Leucine (Biocrates)	Betaine (LC)
	20	Glycerin acid (GC × GC)	Leucine 2 (NMR)	U 3.33 (NMR)^b^

Mean rank from SVM, glmnet, and PLS. Abbreviations in brackets indicate analytical method

*U* unknown, *4-DTA* 4-deoxythreonic acid, *α-KGA* α-ketoglutaric acid, *N-AAA N*-acetylaspartic acid, *4-HPPA* 4-hydroxyphenylpyruvic acid, *PAA para*-acetaminobenzoic acid, *4-HPLA* 4-hydroxyphenyllactic acid, *SM* sphingomyelin, *PC* phosphatidylcholine

^a^Metabolites are identical in the top 20 for sex as well as REE

^b^Metabolites are identical in the top 20 for sex as well as LBM

^c^Tentatively identified using the NIST2011 library solely based on mass spectral similarity

## Discussion

It was unclear whether metabolite profiles may also be used as a suitable tool in the prediction of REE and LBM in healthy humans. Only limited data from metabolomics studies are available so far [20–23]. In the present cross-sectional study, we therefore investigated whether urine as well as plasma metabolite profiles are associated with REE or LBM in healthy subjects under resting conditions. Separately for men and women we could also show in our study that LBM is by far the most important variable to explain REE in healthy subjects (*R*^2^ = 0.77).

Due to the fact that REE and LBM were significantly higher in men than in women (see Table 1), the analysis of metabolite patterns of both REE and LBM were performed separately for men and women. We could not find any metabolite patterns in urine and plasma that enabled a reliable prediction of REE or LBM (see Tables 2, 3). The reason why differences in the metabolically active mass, and also in REE, are not reflected in urine and plasma metabolites cannot be explained by our study results. Our results are in line with previous findings of Frisard et al. and Stretch et al. [24, 44] who also did not find any associations between energy metabolism and metabolite profiles. Frisard et al. examined only markers of oxidative stress in healthy adults [44]. Furthermore, Stretch et al. only investigated 63 urine metabolites in advanced cancer patients [24]. Our results based on a multi-platform metabolomics approach in urine as well as in plasma revealed that also in healthy subjects there is no distinct metabolite profile directly associated with REE.

However, Stretch et al. found that LBM variations could effectively be predicted using urinary metabolites in advanced cancer patients [24]. It should be noted that these analyses were done combined for women and men. To compare our results with those of Stretch et al. [24] and despite the knowledge of the impact of sex on metabolite profiles, analyses on the entire KarMeN study population have been performed. Thus, we could find good prediction accuracies for REE and LBM in plasma as well as urine (*R*^2^ > 0.5; see Supplemental Table 4). In the tertile classified approach, we could demonstrate very good prediction accuracies of 93% for REE and 98% for LBM in plasma as well as urine, respectively (see Supplemental Table 3). These excellent prediction accuracies for healthy subjects confirm the prediction accuracy of 90% recently described by Stretch et al. for lean mass in patients with advanced cancer [24]. However, when we adjusted REE for LBM these prediction accuracies were no longer present (see Supplemental Table 5). We also could show that 13 of the top 20 metabolites in plasma and 11 of the top 20 metabolites in urine are identical and explain differences in sex as well as in REE (see Table 4). We revealed comparable results for LBM, with 12 of the top 20 metabolites in plasma and 14 of the top 20 metabolites in urine being identical for sex, and thus explain differences in sex as well as in LBM (see also Table 4). Therefore, our results support the suggestion that the discrimination of REE or LBM, respectively, seem to be a discrimination of sex. To exclude that the increased sample size when combining data of men and women may improve predicting ability of the model we performed sensitivity analyses on a reduced dataset (50% of men and women, respectively) which resulted in robust prediction accuracies (data not shown). Thus, observed good predictions are likely effects of sex rather than of a higher sample size.

Overall, the focus should be on the metabolite patterns and not on every single metabolite. Nevertheless, associations between various single metabolites and LBM calculated by linear regression models have been shown before [20, 22, 23, 45]. However, these studies did not aim to predict LBM based on metabolite profiles and therefore such data are still missing. We could show a metabolite pattern apparently playing an important role in the prediction of REE or LBM, respectively. Of the metabolites listed in the top 20 mean ranks, many could be identified, some of which have also been described by others: in plasma, creatinine [20, 22], valine [20, 23, 45], leucine [20, 23, 45], glycine, sphingomyelin C18:1, sphingomyelin C16:1, and uric acid [20], in urine, creatinine [24, 46], citrate [46], 4-deoxythreonic acid, 3-indoxylsulfate [24, 46], α-ketoglutaric acid, and gluconic acid could be identified.

Most of the described metabolites not only play an important role in explaining REE or LBM, respectively, but also in distinguishing between men and women. The following metabolites show higher concentrations in men than in women (also higher in high REE or LBM, respectively): creatinine, leucine, 4-deoxythreonic acid, and uric acid. These metabolites are either involved in muscle energy metabolism [13, 16, 18, 47, 48] and known to be determined by the larger muscle mass in men [49], or their function is yet unknown [19, 50]. It is likely that sex differences cause a differential production of muscle-specific metabolites. Other metabolites identified to have lower concentrations in men than in women (also lower in high REE or LBM, respectively) include glycine, sphingomyelin C18:1, citrate, and 3-indoxylsulfate. The reasons for the different metabolite concentrations are not always known. In case of glycine, it could be due to genetic polymorphisms [12, 15, 17], in case of citrate it is largely speculative. Since a further intermediate of the citrate cycle, α-ketoglutaric acid, is also higher in women [13, 16, 18, 19, 47, 51, 52], this may hint at a general difference in citrate cycle turnover between men and women. We cannot exclude that food intake is related to certain metabolites, such as amino acids, which we identified to be associated with LBM. Since plasma samples were collected in the fasted state, we assume that the contribution of dietary factors to, e.g., plasma amino acids is of minor importance. The impact of food intake on metabolite profiles is an important issue and currently under investigation in the KarMeN study.

## Conclusions

We applied targeted and non-targeted metabolomics methods to identify urine as well as plasma metabolites in healthy subjects associated with REE and LBM. As described in the literature and in our study, LBM is the major determinant of REE. The applied machine learning algorithms did not reveal a metabolite pattern predictive for REE or LBM, when analyzing data for men and women, separately. When evaluating data of men and women combined, we were able to predict REE and LBM with high accuracy (> 90%). This, however, may be an effect of sex, since the identified important metabolites for sex show a high degree of overlap with those identified for LBM and REE, respectively. Therefore, under resting conditions REE and LBM have no detectable sex-independent impact on urine and plasma metabolite profiles in the KarMeN study participants. In addition, we conclude that studies applying metabolomics in healthy humans, especially those investigating body composition or energy metabolism, need to consider sex-specific data evaluation.

## Electronic supplementary material

Below is the link to the electronic supplementary material.

Supplementary material 1 (DOCX 29 KB)

## Data Availability

Data are governed by personal data protection rules and may not be shared publicly. Blinded data can be obtained for further research only on the basis of written request to institut.pbe@mri.bund.de and a bilateral data transfer agreement between the data owner (the Max Rubner-Institut) and the requesting institution.

## Abbreviations

BMI: Body mass index
REE: Resting energy expenditure
LBM: Lean body mass
BCM: Body cell mass
IC: Indirect calorimetry
DXA: Dual-energy X-ray absorptiometry
PLS: Partial least squares
SVM: Support vector machine
glmnet: Generalized linear model net
LC: Liquid chromatography
GC: Gas chromatography
GC × GC: Comprehensive two-dimensional gas chromatography
MS: Mass spectrometry
NMR: Nuclear magnetic resonance
CB: Clinical biochemistry
KarMeN: Karlsruhe Metabolomics and Nutrition
TMAO: Trimethylamine-N-oxide
RMSE: Root mean squared error
SM: Sphingomyelin
