# Supplementary material for: Metabolite profiles evaluated, according to sex, do not predict resting energy expenditure and lean body mass in healthy non-obese subjects
Source: Eur J Nutr. 2018 Jul 4;58(6):2207–17. doi: 10.1007/s00394-018-1767-1 (PMC6689277; doi:10.1007/s00394-018-1767-1)
Supplement: Supplementary file 1 — Supplementary material 1 (DOCX 29 KB) [file 394_2018_1767_MOESM1_ESM.docx]

**Supplement**

**Table 1.** Prediction of LBM in the KarMeN study participants (separately for women and men) based on metabolite profiles in plasma and urine using different algorithms.

| Matrix | Algorithm | Men (n=172)  RMSE | R^2^ | Women (n=129)  RMSE | R^2^ |
| --- | --- | --- | --- | --- | --- |
| Plasma | SVM | 6.54 | -0.025 | 3.89 | -0.147 |
|  | PLS | 6.82 | -0.127 | 3.97 | -0.244 |
| Urine | SVM | 6.18 | 0.070 | 3.78 | -0.061 |
|  | PLS | 6.40 | -0.002 | 4.18 | -0.359 |

**Table 2.** Prediction of LBM tertiles in the KarMeN study participants (separately for women and men) based on metabolite profiles in plasma and urine using different algorithms.

|  |  |  |  | Prediction accuracy % |  |
| --- | --- | --- | --- | --- | --- |
| Matrix |  | **Algorithm** | **(total)** | **(low)** | **(high)** |
| Plasma | Men (n=113) | SVM | 50.5 | 44.2 | 57.3 |
|  |  | glmnet | 50.4 | 58.6 | 42.9 |
|  |  | PLS | 50.3 | 50.9 | 50.3 |
|  | Women (n=81) | SVM | 44.8 | 49.7 | 39.5 |
|  |  | glmnet | 45.9 | 48.3 | 43.5 |
|  |  | PLS | 49.9 | 48.8 | 51.0 |
| Urine | Men (n=115) | SVM | 66.2 | 65.5 | 66.7 |
|  |  | glmnet | 64.0 | 64.9 | 63.1 |
|  |  | PLS | 66.7 | 63.4 | 69.9 |
|  | Women (n=86) | SVM | 52.6 | 55.2 | 50.4 |
|  |  | glmnet | 61.5 | 61.3 | 62.3 |
|  |  | PLS | 54.8 | 53.6 | 56.0 |

**Table 3.** Prediction of sex and REE and LBM tertiles in all KarMeN study participants based on metabolite profiles in plasma and urine using different algorithms.

|  |  |  |  | Prediction accuracy % |  |
| --- | --- | --- | --- | --- | --- |
| Matrix |  | **Algorithm** | **(total)** | **(low or men)** | **(high or women)** |
| Plasma | REE (n=194) | SVM | 93.4 | 92.6 | 94.2 |
|  |  | glmnet | 90.1 | 88.7 | 91.4 |
|  |  | PLS | 93.2 | 92.0 | 94.4 |
|  | LBM (n=194) | SVM | 98.3 | 98.2 | 98.3 |
|  |  | glmnet | 95.6 | 97.2 | 93.9 |
|  |  | PLS | 97,6 | 97.6 | 97.7 |
|  | Sex (n=291) | SVM | 98,1 | 98.6 | 97.5 |
|  |  | glmnet | 97.7 | 98.0 | 97.2 |
|  |  | PLS | 96.7 | 96.6 | 96.9 |
| Urine | REE (n=201) | SVM | 91.9 | 90.3 | 93.6 |
|  |  | glmnet | 92.4 | 90.5 | 94.2 |
|  |  | PLS | 93.2 | 91.6 | 94.8 |
|  | LBM (n=201) | SVM | 92.7 | 90.5 | 94.8 |
|  |  | glmnet | 92.7 | 91.7 | 93.8 |
|  |  | PLS | 93.0 | 91.1 | 94.8 |
|  | Sex (n=301) | SVM | 93.4 | 94.5 | 91.8 |
|  |  | glmnet | 92.4 | 94.9 | 89.2 |
|  |  | PLS | 92.6 | 95.6 | 88.7 |

**Table 4.** Prediction of REE and LBM in all KarMeN study participants based on metabolite profiles in plasma and urine using different algorithms.

| Matrix | Algorithm | REE (n=301)  RMSE | R^2^ | LBM (n=301)  RMSE | R^2^ |
| --- | --- | --- | --- | --- | --- |
| Plasma | SVM | 173 | 0.515 | 6.60 | 0.586 |
|  | glmnet | 169 | 0.536 | NA | NA |
|  | PLS | 173 | 0.515 | 6.67 | 0.578 |
| Urine | SVM | 178 | 0.484 | 6.59 | 0.588 |
|  | PLS | 175 | 0.503 | 6.72 | 0.571 |

**Table 5.** Prediction of REE adjusted for LBM (REE_adj_) in all KarMeN study participants based on metabolite profiles in plasma and urine using different algorithms.

| Matrix | Algorithm | REE_adj_ (n=301)  RMSE | R^2^ |  |  |
| --- | --- | --- | --- | --- | --- |
| Plasma | SVM | 118 | 0.038 |  |  |
|  | glmnet | 116 | 0.065 |  |  |
|  | PLS | 120 | -0.003 |  |  |
| Urine | SVM  glmnet | 121  121 | -0.007  -0.005 |  |  |
|  | PLS | 125 | -0.085 |  |  |

**Table 6.** Most important metabolites in predicting REE (tertile classified approach) in urine: ranks from SVM, glmnet, and PLS.

| **Metabolite** | **SVM rank** | **glmnet rank** | **PLS rank** |
| --- | --- | --- | --- |
| 1. U 2.19 (NMR) | 1 | 2 | 1 |
| 1. citrate 2 (NMR) | 6 | 3 | 2 |
| 1. 4-DTA (GCxGC) | 9 | 1 | 4 |
| 1. U 7.57 (NMR) | 2 | 6 | 6 |
| 1. citrate 1 (NMR) | 7 | 5 | 3 |
| 1. creatinine 2 (NMR) | 16 | 4 | 5 |
| 1. U 05 (GC) | 3 | 12 | 11 |
